# Development of an In Vitro Membrane Model to Study the Function of EsxAB Heterodimer and Establish the Role of EsxB in Membrane Permeabilizing Activity of *Mycobacterium tuberculosis*

**DOI:** 10.3390/pathogens9121015

**Published:** 2020-12-02

**Authors:** Salvador Vazquez Reyes, Supriyo Ray, Javier Aguilera, Jianjun Sun

**Affiliations:** 1 Department of Biological Sciences, University of Texas at El Paso, 500 West University Avenue, El Paso, TX 79968, USA; svazquezre@miners.utep.edu (S.V.R.); jaguilera5@miners.utep.edu (J.A.); 2 Border Biomedical Research Center at University of Texas at El Paso, 500 West University Avenue, El Paso, TX 79968, USA; 3 Department of Chemistry & Biochemistry, University of Texas at El Paso, 500 West University Avenue, El Paso, TX 79968, USA; 4 Department of Natural Sciences, Bowie State University, 14000 Jericho Park Rd, Bowie, MD 20715, USA

**Keywords:** *Mycobacterium tuberculosis*, EsxA, EsxB, ESAT-6, CFP-10, membrane permeabilizing activity

## Abstract

EsxA and EsxB are secreted as a heterodimer and have been shown to play critical roles in phagosome rupture and translocation of *Mycobacterium tuberculosis* into the cytosol. Recent in vitro studies have suggested that the EsxAB heterodimer is dissociated upon acidification, which might allow EsxA insertion into lipid membranes. While the membrane permeabilizing activity (MPA) of EsxA has been well characterized in liposomes composed of di-oleoyl-phosphatidylcholine (DOPC), the MPA of EsxAB heterodimer has not been detected through in vitro assays due to its negligible activity with DOPC liposomes. In this study, we established a new in vitro membrane assay to test the MPA activity of N-terminal acetylated EsxA (N-EsxA). We established that a dose-dependent increase in anionic charged lipids enhances the MPA of N-EsxA. The MPA of both N-EsxA and EsxAB were significantly increased with this new liposome system and made it possible to characterize the MPA of EsxAB in more physiologically-relevant conditions. We tested, for the first time, the effect of temperature on the MPA of N-EsxA and EsxAB in this new system. Interestingly, the MPA of N-EsxA was lower at 37 °C than at RT, and on the contrary, the MPA of EsxAB was higher at 37 °C than at RT. Surprisingly, after incubation at 37 °C, the MPA of N-EsxA continuously decreased over time, while MPA of EsxAB remained stable, suggesting EsxB plays a key role in stabilizing N-EsxA to preserve its MPA at 37 °C. In summary, this study established a new in vitro model system that characterizes the MPA of EsxAB and the role of EsxB at physiological-relevant conditions.

## 1. Introduction 

Tuberculosis (TB) is a leading infectious disease in the world that accounts for tens of millions of new cases and more than one million deaths per year [1]. *Mycobacterium tuberculosis* (Mtb), once inhaled, reaches alveoli where it comes into contact with dendritic cells [2] and macrophages [3]. The macrophages engulf Mtb into phagosomes. Under normal circumstances, the phagosome matures into a phago-lysosome by fusing with a lysosome, which causes a drop in pH and the release of reactive oxygen intermediates that digest mycobacteria. However, Mtb either prevents or avoids phagosome maturation via different mechanisms such as phagosome rupture, pH regulation [4], and phagosome–lysosome fusion arrest [5,6,7]. As a result, Mtb is translocated from the phagosome [8,9] into the cytosol, thereby avoiding digestion in the phago-lysosome. Mtb translocation activity is attributed to the RD1 locus that codes for a type VII secretion system and its substrates EsxA (ESAT-6) and EsxB (CFP-10). These proteins have been described as essential virulence factors, as their absence attenuates mycobacterial virulence [8,10,11,12,13,14,15,16].

EsxA and EsxB form a heterodimer (EsxAB) that is co-secreted when Mtb is phagocytized by macrophages [12,15,17]. In previous in vitro studies, EsxA protein has been shown to undergo significant conformational changes and exhibits strong membrane permeabilization activity (MPA) upon acidification [18]. By contrast, EsxB does not have MPA and does not undergo any significant conformational change [9,18,19,20,21]. Though EsxB may not play any role in membrane permeabilization, it is required for EsxA secretion and may act as a chaperone [17,19,22,23,24]. The EsxAB membrane-permeabilizing mechanism has not been described yet and its role in mycobacterial phagosome escape and cytosolic translocation is still under debate. Current studies have suggested a model that the EsxAB heterodimer is dissociated at low pH to allow EsxA to interact and permeabilize the membrane [9,18,19,24]. However, macrophage infection assays with *M. marinum* indicated that phagosomal pH decrease did not trigger EsxAB-mediated membrane disruption [21], and there may be other cellular or bacterial factors involved. The absence of an efficient in vitro model to study EsxAB MPA makes the task more challenging. While the MPA of EsxA has been well characterized in liposomes composed of DOPC, a neutral fluidic lipid, the MPA of EsxAB has been poorly studied due to its extremely low activity with the DOPC liposomes. The role of EsxB in MPA has been speculative, and there is no in vitro experimental evidence to convincingly establish its role in the permeabilizing activity.

Our recent study has found that the MPA of EsxA can be significantly affected by the physical characteristics of lipid membranes, such as fluidity and charge [25]. Fluidity (the presence of unsaturated acyl chain) is required for EsxA to permeabilize the membrane, and the presence of negatively charged lipids enhances the MPA of EsxA [25]. The EsxA used in that study was purified from *E. coli* and lacked post-translational modifications. Our recent studies showed that EsxA derived from *M. smegmatis* is N-terminally acetylated [24]. Inspired by these findings, in this study, we used *M. smegmatis-*derived N-terminally acetylated EsxA for our studies. In our previous study using *E. coli*-derived EsxA, we showed that MPA activity was highly efficient with a physiologically-relevant liposome model (POPC:POPG) compared to DOPC neutral charged membrane lipids. In this study, we optimized our membrane model by titrating the anionic charged lipids (e.g., POPG) to determine their dose-dependent effects on the MPA of N-EsxA.

Next, we found that the optimized liposome system significantly enhanced the MPA of EsxAB to a level that has never been reached before. It is important to note, so far, that there are very few studies that establish the role of EsxB in MPA, and there are no in vitro assays that test the MPA of EsxAB. This study establishes an effective membrane model for the characterization of the MPA of EsxAB.

Thus far, most membrane permeabilization in vitro assays using lipid vesicles with EsxA and EsxAB were done between 20 °C and 33 °C [9,18,19,20,21,24,26]. Because the melting temperature of EsxA is 35 °C, EsxA retains more than 50% of its native structure at 33 °C, while at 37 °C EsxA loses more than 50% of its native structure and become unstable [25]. Hence, it is imperative to re-investigate the MPA of EsxA and the role of EsxB in membrane permeabilization at 37 °C. In comparison, EsxAB is more thermodynamically stable at 37 °C with a Tm at ~53 °C [22]. This suggests that EsxB plays a larger role in membrane permeabilization at 37 °C. To date, there is no effective membrane permeabilization in vitro assay that captures the role of EsxB at physiological temperature.

## 2. Methods and Procedures

### 2.1. Lipids

The lipids 1-palmitoyl-2-oleoyl-sn-glycero-3-phosphatidylcholine (POPC), 1-palmitoyl-2-oleoyl-sn-glycero-3-phosphatidylglycerol (POPG), 1,2-dioleoyl-sn-glycero-3-phosphocholine (DOPC), and 1,2-dioleoyl-sn-glycero-3- phosphatidylglycerol (DOPG) were purchased from Avanti Polar Lipids (Alabaster, AL, USA).

### 2.2. EsxA and EsxAB Heterodimer Expression and Purification

The pMyNT(EsxA:EsxB) plasmid was a generous gift from Dr. Matthias Wilmanns. Expression of this plasmid in *M. smegmatis* produces EsxAB heterodimer (from *M. tuberculosis)* with a His_6_ tag at the N-terminus of EsxB. The heterodimer expressed in *M. smegmatis* also contains mycobacterial native post-translational modification (N^a^-acetylation) that was described in a previous study [24]. The pMyNT(EsxA(S35C):EsxB) plasmid was generated by site-directed mutagenesis described in a previous publication [26]. Protein purification was performed as previously described [25].

### 2.3. EsxAB Heterodimer Separation

The EsxAB heterodimer was separated by using a 6 M guanidine solution. The EsxAB-containing solution was concentrated to 1 mL and injected into a 5-mL His trap column, which was pre-equilibrated with guanidine solubilization buffer (25 mM NaH_2_PO_4_, 150 mM NaCl, 10 mM imidazole, and 6 M guanidine, pH 6.8). The His_6_-tagged EsxB bound to the column, while EsxA was collected in the flow through. The collected flow-through fractions were concentrated and extensively dialyzed in a 3-kDa cutoff membrane in a dialysis buffer (25 mM NaH_2_PO_4_, 100 mM NaCl, and 1 mM EDTA pH 7.4), in which the denatured EsxA was refolded. The EsxB in the column was eluted with a linear gradient of (10–100%) of an elution buffer (25 mM NaH_2_PO_4_, 150 mM NaCl, 500 mM imidazole, and 6 M guanidine). The eluted fractions were collected and extensively dialyzed in a 3-kDa cutoff membrane in the dialysis buffer (25 mM NaH_2_PO_4_, 100 mM NaCl, and 1 mM EDTA pH 7.4).

### 2.4. Liposome Preparation

In most of our previous studies, characterization of EsxA MPA was conducted in large unilamellar vesicles (LUVs) made of DOPC (1,2-dioleoyl-sn-glycero-3-phosphatidylcholine), which is a neutral lipid with both acyl unsaturated chains [19,20,25,26]. This lipid has been widely used to characterize pore-forming proteins due to their ability to form fluid membranes [27,28,29]. However, we recently found that both membrane fluidity and charge affected EsxA MPA. Given sufficient fluidity, negative charge significantly enhanced EsxA MPA. Thus, in this study, a mixture of 1-palmitoyl-2-oleoyl-sn-glycero-3-phosphatidylcholine (POPC) and 1-palmitoyl-2-oleoyl-sn-glycero-3-phosphatidylglycerol (POPG) were used to prepare liposomes to mimic the inner leaflet of phagosomes [21,30,31,32,33,34,35,36,37,38,39,40,41]. As controls, the DOPC/DOPG liposomes were used to compare with the new liposomes [25]. The final lipid concentration was 0.8 mM, and the size of each LUV was 200 nm in diameter. The PC and PG lipids were weighed and mixed to produce 0.8 mM liposomes with the relative PC/PG molar concentration of 100%/0%, 95%/5%, 90%/10%, 80%/20%, and 60%/40%.

### 2.5. Time Lapse Intensity Measurement of EsxA Membrane Permeabilization by ANTS/DPX Fluorescence Dequenching

EsxA MPA was measured in real-time by using the 8-aminonapthalene-1,3,6-trisulfonic acid (ANTS)/*p*-xylene-bis-pyridinium bromide(DPX) fluorescence dequenching assay, as described in previous publications [19,20,26]. Furthermore, we evaluated the effects of different lipid compositions and temperatures on the MPA of EsxAB heterodimer. The lipids were solubilized in 1 mL of chloroform:methanol (3:2) solution and dried in nitrogen gas. ANTS and DPX were weighed and added to a final concentration of 10 mM. The mixture of lipids, ANTS, and DPX were resuspended in 1 mL of 5 mM HEPES, pH 7.4. The samples were frozen and thawed for 6 cycles. After this, the mixture was extruded through a 200 nm pore size membrane 23 times (back and forth) using an Avanti Polar Lipids, Inc. mini extruder set (cat no. 610000). The excess ANTS and DPX were removed by passing the sample through a 5 mL desalting column. The liposome fractions were collected and stored at 4 °C until use.

The ANTS/DPX fluorescence dequenching was measured in an ISS-K2 multiphase frequency and modulation fluorometer with excitation at 380 nm, and emissions were recorded at 520 nm. The liposomes (800 µM) containing ANTS/DPX were diluted into 1.35 mL of gel filtration buffer at pH 7.4, which had 4.76 μM of EsxA or EsxAB. The liposomes and proteins were incubated for 15 min at RT or 37 °C in the fluorometer. Following incubation, the fluorescence base line of the samples was monitored for 30 sec, and 1/10 volume (150 µL) of 1 M sodium acetate (pH 4.0) was injected into the cuvette to decrease the pH to 4.0. The samples were continuously stirred during the experiment and crossed polarized on excitation and emission beams, and a 515 nm long-pass filter was used to reduce the background scatter. The fold changes of ANTS fluorescence with the samples were calculated by using EsxA in DOPC as a control. The rates of ANTS fluorescence dequenching were calculated in GraphPad Prism by curve fitting plateau followed by a one phase association $y=IF(x\;<,y_{o},y_{0}+\left(Plateau-y_{0}\right)*\left(1-e^{\left(x-x_{o}\right)}\right))$. One-way ANOVA was used for statistical significance analysis.

### 2.6. Time-Lapse Intensity Measurement of NBD Fluorescence for EsxA Membrane Insertion

*N*,*N*′-Dimethyl-*N*-(Iodoacetyl)-*N*′-(7-Nitrobenz-2-Oxa-1,3-Diazol-4-yl)Ethylenediamine (IANBD) is a fluorophore that increases its fluorescence as it transitions from a hydrophilic environment to a hydrophobic environment. In our previous publications, the NBD-labeled EsxA(S35C) was successfully used to directly confirm the insertion of EsxA into the liposome membranes [20,25,26,29,42]. In brief, the purified EsxA(S35C) was reduced with dithiothreitol (DTT) on ice for 20 min with a DTT/protein ratio of 250:1. Then DTT was removed from the sample by passing the sample through a desalting column with a buffer (50 mM HEPES, 150 mM NaCl, 50 mM sodium acetate pH 8). Then, IANBD was added in 20x molar excess and incubated at RT for 2 h in the dark. Finally, the excess of NBD was removed by passing the sample through a desalting column. The labeling efficiency for EsxA(S35C) was ~75%. The NBD fluorescence was measured in the ISS-K2 multiphase frequency and modulation fluorometer with excitation at 488 nm and emission at 544 nm. The NBD-labeled EsxA(S35C) or EsxA(S35C)-B heterodimer (1.36 μM) were incubated with 800 μM of the liposomes at 4 °C for 30 min in the buffer (20 mM TrisHCl and 100 mM NaCl, pH 7.4) as previously described [25]. The liposome/protein mixtures were incubated for 15 min at RT or 37 °C in the fluorometer with continuous stirring. Excitation and emission beams were cross polarized, and a 520 nm long-pass filter was used to reduce the back-ground scatter. After the addition of 1/10 volume of 1 M sodium acetate (pH 4.0), the NBD fluorescence emission was monitored. The fluorescence fold of change was calculated for each sample by using the sample EsxA with DOPC liposome as a control. 

### 2.7. EsxB Stabilization Effect on EsxA at 37 °C

Time lapse intensity measurement of ANTS/DPX dequenching was used to evaluate the stabilization effect of EsxB on EsxA structure at 37 °C. EsxA or EsxAB were pre-incubated at 37 °C for the increasing time lapses (from 0 to 30 min) before fluorescence measurement. The EsxA membrane disruption was triggered by acidification as described above. The fluorescence fold of change was calculated as described above. The experiment was done in triplicate and replicated at least three times for the statistical analysis. Two-way ANOVA with a multiple comparison test was used to compare EsxA and EsxAB after incubation at 37 °C for different amounts of time. Then, a multiple comparison Tukey–Kramer test was used to find significant differences for each group. 

## 3. Results

### 3.1. Incorporation of Negatively Charged Lipids Increased N^α^-Acetylated EsxA (N-EsxA) Membrane Permeabilization

In our previous studies, N-EsxA MPA was characterized with the liposomes made of DOPC, an unsaturated lipid that confers membrane fluidity [19,20,26]. Recently, we have shown that while membrane fluidity is required for EsxA to permeabilize the membranes, incorporation of negatively charged lipids at the concentrations similar to phago-endosomal membranes increases EsxA membrane permeabilization [25,43]. Here, we systematically titrated the concentrations of PG lipids relative to PC lipids to determine the effects of the PG lipids on the MPA of N-EsxA.

**Figure 1:**
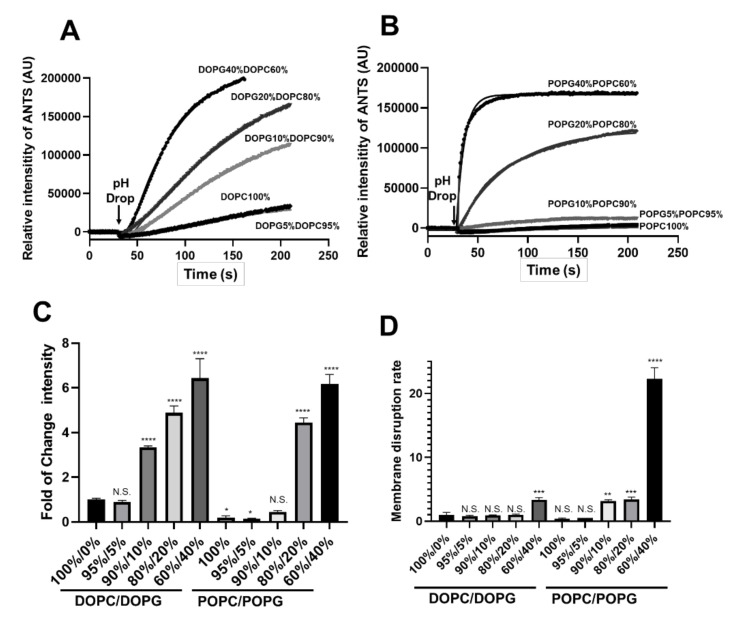
Incorporation of the negatively charged PG into the PC-made liposome enhanced EsxA-membrane permeabilizing activity. (**A**) The liposomes were prepared with DOPC and DOPG at the indicated percentages. The liposomes were pre-incubated with the purified N-EsxA at pH 7 at RT for 15 min; they were then transferred to a cuvette in a fluorometer, and ANTS fluorescence was monitored in real-time. N-EsxA-mediated membrane permeabilization was triggered by the addition of 1/10 volume of 1 M sodium acetate. (**B**) Similarly, the N-EsxA-mediated membrane permeabilization was performed on the POPC/POPG liposomes at the indicated percentage using the ANTS/DPX assay. (**C**) Relative fold change of ANTS fluorescence intensity in the DOPC/DOPG and POPC/POPG liposomes. (**D**) The curves of ANTS fluorescence intensity were fit into a nonlinear regression (plateau followed by a one-phase association), and the initial rate of membrane disruption was calculated. The rate of N-EsxA DOPC at RT was set as a control. SD is represented in the error bars. One-way ANOVA with multiple comparison test was performed for (**C**,**D**) (*p* < 0.05) *, (*p* < 0.01) **, (*p* < 0.005) ***, (*p* < 0.0001) ****. The experiment was done in triplicate and replicated at least three times for the statistical analysis.

We first used the ANTS/DPX fluorescence dequenching assay, an approach for measuring membrane permeabilization, to test the liposomes made of DOPC/DOPG at various molar ratios (%), namely 100/0, 95/5, 90/10, 80/20, and 60/40, respectively. Like in our previous studies with EsxA, the MPA of N-EsxA was significantly enhanced by DOPG in a concentration-dependent manner. Incorporation of DOPG at 10%, 20%, and 40% concentration yielded 3.32, 4.82, and 6.4 times the increase in membrane permeabilization, respectively (Figure 1A,C).

Next, we tested the POPC/POPG liposomes at various molar ratios (%), namely 100/0, 95/5, 90/10, 80/20, and 60/40, respectively. Similarly, the membrane permeabilization was increased in a POPG concentration-dependent manner (Figure 1B,C). We noticed that the activity in pure POPC liposome was lower than pure DOPC, which is consistent with the previous study because DOPC is more fluid than POPC [25]. In the liposomes with POPC/POPG at 95/5 and 90/10, the membrane permeabilization by EsxA had a slight increase relative to pure POPC, but it was sharply increased with liposomes POPC/POPG at 80/20 and 60/40, respectively. It is important to mention that POPC/POPG at 90/10, 80/20, and 60/40 showed a 3.1, 3.4, and 22.2 times of membrane disruption rate, respectively, compared to the DOPC/DOPG 100/0 formulation (Figure 1A,D). Thus, N-EsxA exhibits a much higher rate of membrane permeabilization with the POPC/POPG liposomes, similar to EsxA.

### 3.2. The New Liposome System Was Validated by NBD Fluorescence

**Figure 2:**
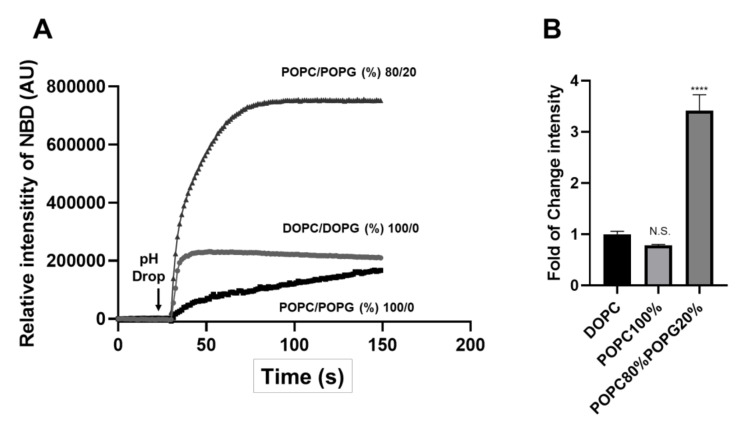
EsxA membrane insertion was significantly enhanced in the liposome containing POPG. (**A**) DOPC/DOPG (%) 100/0, POPC/POPG (%) 100/0, and 80/20 liposomes were pre-incubated with the NBD-labeled EsxA(S35C) in pH 7 at RT for 15 min. Then, they were transferred to a cuvette in a fluorometer, and NBD fluorescence intensity was monitored in real-time. EsxA membrane insertion was triggered by the addition of 1/10 volume of 1M sodium acetate. (**B**) Relative fold of change of NBD fluorescence intensity in DOPC, POPC, and POPC80%POPG20% liposomes. SD is represented in the error bars. One-way ANOVA with multiple comparison test was performed for (**B**) (*p* < 0.0001) ****. The experiment was done in triplicate and replicated at least three times for the statistical analysis.

In order to validate the results obtained in the new liposome system, we used the NBD-labeled EsxA to test the physical insertion of EsxA into the liposomal membrane as an independent approach (Figure 2). The membrane insertion of EsxA was significantly increased with the liposomes containing POPC/POPG (%), compared to the pure DOPC and POPC liposomes (Figure 2A,B). The results of membrane insertion measured by NBD fluorescence are consistent with the results obtained in the ANTS/DPX membrane permeabilization assays (Figure 1).

In summary, we used two independent assays (ANTS/DPX and NBD) and tested the MPA of N-EsxA in the new liposome systems made of either DOPC/DOPG or POPC/POPG. We found that incorporation of the negatively charged PG lipids greatly enhanced the MPA of N-EsxA, and it particularly exhibited higher MPA with the POPC/POPG liposomes.

### 3.3. Characterization of the Heterodimer’s MPA in the PC/PG Liposomes

N-EsxA and EsxB are co-expressed and co-secreted as a heterodimer after Mtb is phagocytized by the macrophage [15]. Current studies have suggested the heterodimer needs to be dissociated prior to N-EsxA membrane insertion [18,19]. We recently showed that N-terminal acetylation is required for the dissociation of these two proteins [19,24]. However, the characterization of the heterodimer MPA had been a challenge with the DOPC liposomes due to low activity detected by ANTS/DPX assay. Here, we used the POPC/POPG (%) 80/20 liposomes to test the heterodimer’s MPA.

**Figure 3:**
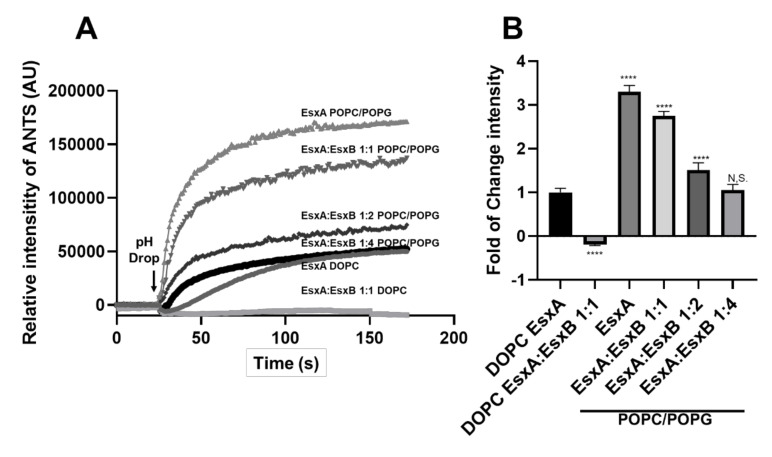
The N-EsxA membrane permeabilization activity was inhibited by EsxB in a dose-dependent manner. (**A**) The purified N-EsxA was pre-incubated with EsxB at various molar ratios (1:1, 1:2, 1:4) at 4 °C overnight. Then, N-EsxA and EsxA:EsxB mixtures were incubated with the DOPC liposome or POPC/POPG(%) 80/20 liposome (POPC/POPG) at pH 7 at RT for 15 min and then transferred to a cuvette in a fluorometer. The ANTS fluorescence was monitored in real-time. N-EsxA-mediated membrane permeabilization was triggered by the addition of 1/10 volume of 1 M sodium acetate. (**B**) Relative fold of change of ANTS fluorescence intensity using EsxA in DOPC/DOPG (%) 100/0 as a reference (set as 1). SD is represented in the error bars. One-way ANOVA with multiple comparison test was performed for (**B**) (*p* < 0.0001) ****. The experiment was done in triplicate and replicated at least three times for the statistical analysis.

The membrane permeabilization activity (fold change of ANTS intensity) of N-EsxA with the DOPC liposomes was set as the control. EsxA:EsxB (1:1) had nearly undetectable membrane permeabilization (Figure 3). Interestingly, with the POPC/POPG liposomes, EsxA:EsxB (1:1) had a significant increase in membrane permeabilization, which was about three-fold of the control. The displayed MPA in POPC/POPG liposomes was high enough to provide us with a “big window” to titrate the inhibitory effect of EsxB on EsxA MPA. As expected, EsxB inhibited the MPA of N-EsxA in a dose-dependent manner, indicating that heterodimer dissociation is required for N-EsxA to permeabilize the membranes (Figure 3), which is consistent with our previous findings [24]. It is important to note that the heterodimer EsxAB was comprised of N-EsxA purified from *M. smegmatis*.

### 3.4. EsxAB is More Stable at 37 °C Than N-EsxA

**Figure 4:**
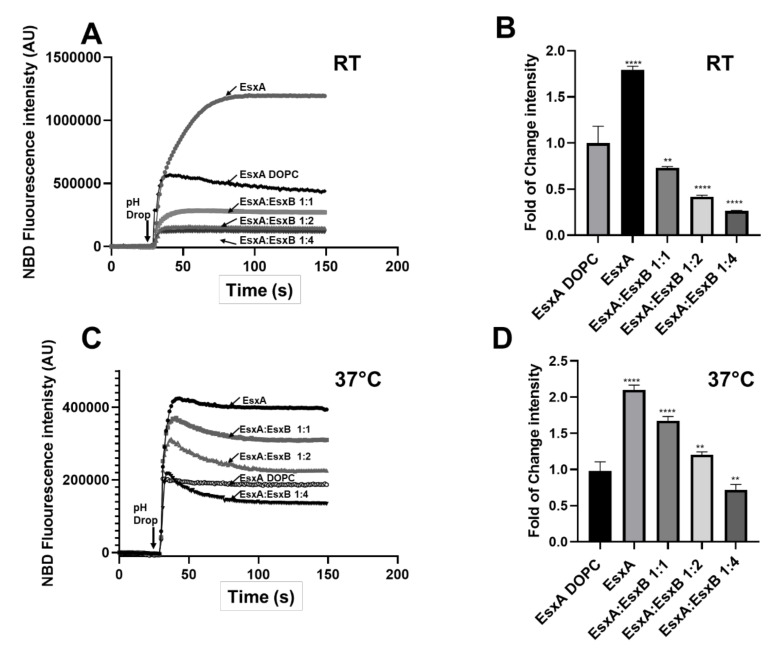
N-EsxA membrane insertion was inhibited by EsxB, but the inhibition was attenuated at physiological temperature. (**A**) The NBD-labeled EsxA(S35C) and EsxB were pre-incubated with the indicated molar ratios at 4 °C overnight. The proteins were incubated with DOPC liposome or POPC/POPG(%) 80/20 liposome at pH 7 and RT for 15 min. They were transferred to a cuvette in a fluorometer, and NBD fluorescence was monitored in real-time. EsxA(S35C) membrane insertion was triggered by the addition of a 1/10 volume of 1 M sodium acetate. (**B**) The relative fold change of NBD fluorescence intensity of the samples was calculated using the NBD intensity of EsxA(S35C) on DOPC/DOPG (%) 100/0 liposome as the reference. (**C**) The NBD-labeled EsxA(S35C) and EsxB were pre-incubated at the indicated molar ratios at 4 °C overnight. Then the proteins were incubated with DOPC/DOPG (%) 100/0 liposome or POPC/POPG (%) 80/20 liposome at pH 7 and 37 °C for 15 min. They were transferred to a cuvette in a fluorometer, and NBD fluorescence was monitored in real-time at 37 °C. EsxA(S35C) membrane insertion was triggered by the addition of a 1/10 volume of 1 M sodium acetate. (**D**) The relative fold of change of NBD fluorescence intensity of the samples was calculated using the NBD intensity of EsxA(S35C) on DOPC liposome as the reference. SD is represented in the error bars. One-way ANOVA with multiple comparison test was performed for (**B**,**D**) (*p* < 0.001) **, (*p* < 0.0001) ****. The experiment was done in triplicate and replicated at least three times for the statistical analysis.

Subsequently, we set out to confirm the results in membrane insertion of N-EsxA and EsxAB. We incubated N-EsxA and N-EsxA:EsxB (1:1, 1:2, and 1:4 ratio) at RT and 37 °C. At RT, N-EsxA MPA was significantly inhibited by EsxB in a dose-dependent manner. This was shown as a sharp drop even at a 1:1 ratio of N-EsxA:EsxB, where the MPA was inhibited by more than 50% (Figure 4A,B). At 37 °C, the inhibition of EsxB on N-EsxA MPA was less significant, evidenced by a slow dose-dependent decrease (Figure 4C,D). The result suggests that EsxAB is more active at 37 °C than at RT. 

**Figure 5:**
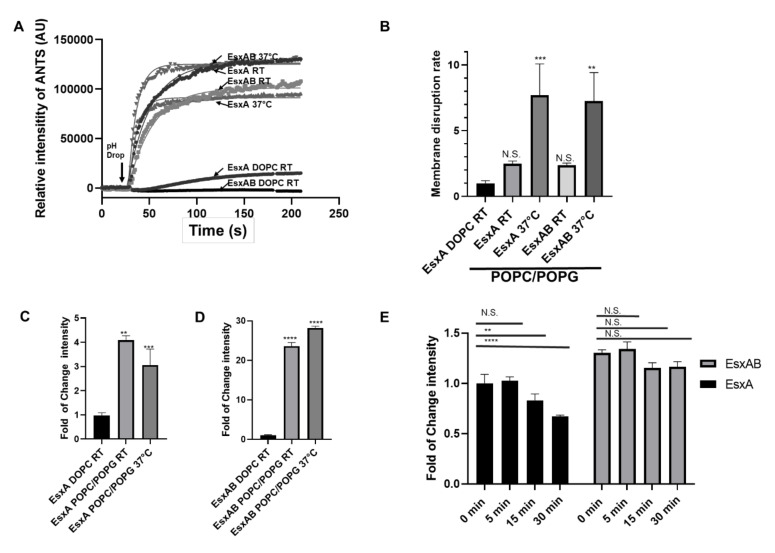
EsxAB is more efficient in membrane permeabilization at the physiological temperature of 37 °C. (**A**) N-EsxA and EsxAB were preincubated with DOPC/DOPG (%) 100/0 liposome and POPC/POPG(%) 80/20 liposome (POPC/POPG) at either RT or 37 °C for 15 min. The ANTS fluorescence was monitored in real-time in a cuvette in a fluorometer. The N-EsxA-mediated membrane permeabilization was triggered by the addition of 1/10 volume of 1 M sodium acetate. (**B**) The curves of ANTS fluorescence intensity were fit into a nonlinear regression (plateau followed by one-phase association), and the initial rate of membrane disruption was calculated. The rate of N-EsxA DOPC/DOPG (%) 100/0 at RT was set as a control. (**C**) Relative fold of change of ANTS intensity of N-EsxA at RT and 37 °C compared with that of EsxA DOPC/DOPG (%) 100/0 at RT. (**D**) Relative fold of change of ANTS intensity of EsxAB at RT and 37 °C compared with EsxAB DOPC/DOPG (%) 100/0 at RT. (**E**) N-EsxA and EsxAB were incubated with the POPC/POPG (%) 80/20 liposomes at pH 7 at 37 °C according to each time point, then membrane disruption was triggered, and ANTS fluorescence was recorded as described in (**A**). Relative folds changes of ANTS intensity of N-EsxA and EsxAB were calculated for each time point. A 0 min time point was used as a comparison reference. SD is represented in the error bars. One-way ANOVA was performed for (**B**–**E**) (*p* < 0.05) **, (*p* < 0.00012) ***, (*p* < 0.0001) ****. Two-Way ANOVA with a Tukey multiple comparison test was performed for (**E**) (*p* < 0.001) **, (*p* < 0.0001) ****. The experiment was done in triplicate and replicated at least three times for the statistical analysis.

In our previous publication, CD analysis showed that the T_m_ of EsxA was 35 °C and the T_m_ of EsxAB was 53 °C [22], meaning that at 37 °C, EsxA will have less than 50% of its native structure, in contrast to EsxAB, which will have a conserved native structure. It is not clear how the temperature will affect the MPA of N-EsxA and EsxAB. Thus, we tested the MPA of N-EsxA and EsxAB at RT (22 °C) and 37 °C. For both N-EsxA and EsxAB with the POPC/POPG liposomes, the rate of membrane permeabilization at 37 °C was much higher than that at RT (Figure 5A,B). Interestingly, however, the fold change of ANTS fluorescence of N-EsxA at RT was lower than at 37 °C, while the fold change of fluorescence intensity of EsxAB at RT was lower than 37 °C (Figure 5C,D). This suggests that while N-EsxA and EsxAB had similar initial rates of membrane permeabilization, EsxAB was more stable and hence remained active longer than N-EsxA in membrane permeabilization at 37 °C.

To test this hypothesis, N-EsxA and EsxAB were pre-incubated at 37 °C at varying time frames (0, 5, 15, and 30 min) and were then tested for membrane permeabilization. The result showed that N-EsxA MPA was significantly decreased as the incubation time at 37 °C increased, while EsxAB MAP remained constant over time (Figure 5E). The data suggests that at 37 °C, N-EsxA was progressively unfolded in solution and lost MPA over time, and EsxB played a role in stabilizing N-EsxA at 37 °C to preserve its MPA until acidification.

## 4. Discussion

The MPA of EsxA has been extensively characterized [8,19,20]. We have shown that N-EsxA from Mtb exhibits a unique MPA that is absent in its ortholog from *M. smegmatis* [19]. We obtained direct evidence that N-EsxA inserts into the liposomal membrane and forms a membrane-spanning structure [20]. Most recently, we have shown that the EsxA protein produced in *M. smegmatis* instead of *E. coli* contains mycobacterial-specific N-terminal acetylation, which is required for membrane permeabilization. Once Thr2 of EsxA is acetylated, it creates a dragging force that pulls away EsxB from EsxA and allows heterodimer dissociation in low pH [24], an event critical prior to membrane disruption [9,18,19,24]. Numerous studies have implicated that EsxAB ruptures the phagosome membrane and assists mycobacterial escape into the cytosol [8,9,12,18]. In the current DOPC liposome model, however, EsxAB exhibits very low MPA, which suggests DOPC liposome may not be suitable for the characterization of EsxAB MPA. The lack of a good membrane model has hindered our understanding of the molecular mechanism of EsxAB-mediated virulence. In this study, we tested a new liposome system made of PC/PG lipids that greatly enhanced membrane permeabilization by EsxAB. The PC/PG liposome is a capable tool to study EsxAB membrane permeabilization in a physiological-like condition. The use of PC and PG lipids instead of phosphatidylserine makes this model accessible to multiple study groups. However, the physiological characteristics of the phagosome membranes are conserved by using these lipids. We expect that the PC/PG liposome model will contribute to elucidate the mechanism of EsxAB mediated permeabilization into membranes.

Our previous studies have shown that the physical characteristics of membrane lipids can modulate N-EsxA interaction with the membranes [25]. We also identified new membrane models that effectively capture membrane permeabilization by N-EsxA at room temperature. We showed that N-EsxA has a much higher efficiency of membrane permeabilization in the presence of POPC/POPG at a 4:1 ratio, which closely mimics the inner leaflet of the plasma membrane in terms of fluidity and charge. In the present study, we systematically characterized the effects of the incorporation of negatively charged PG lipids into the PC liposome on the MPA of N-EsxA and EsxAB (Figure 1 and Figure 2). Furthermore, the EsxB titration experiment showed that EsxB inhibited N-EsxA membrane permeabilization in a dose-dependent manner (Figure 3). The result is consistent with the previous findings that EsxA needs to dissociate from EsxB prior to the membrane disruption [19,24].

After establishing the new liposome system, we tested the effects of temperature on membrane permeabilization. Previous studies have shown that EsxB has no specific membrane interaction, and EsxAB needs to be dissociated to allow EsxA insert into the membrane [9,18,19,24]. Here, we acquired an incremental understanding of N-EsxA and EsxB interaction at 37 °C. Previous membrane permeabilization assays were performed either using the pure DOPC liposome at RT [19,24], or sheep RBCs (red blood cells) that were incubated at 33 °C [9,18]. Thermal stability of EsxA (S35C) through CD analysis suggests that EsxA has a melting temperature around 35 °C; thus, at RT or 33 °C, EsxA retains more than half of its native structure. This allows the protein to interact efficiently with the membrane without requiring EsxB, but EsxA loses more than 50% of its native structure at 37 °C. Earlier studies have shown that EsxAB has a melting temperature around 53 °C; hence, it is more stable at 37 °C than EsxA [22]. Our experiments showed that N-EsxA by itself induced lower membrane permeabilization than the heterodimer at 37 °C (Figure 4 and Figure 5). For the first time, this experiment captured the role of EsxB in stabilizing the structure of N-EsxA at 37 °C to achieve optimum efficiency to permeabilize the membrane. EsxB assists in the maintenance of N-EsxA structure as a chaperone that increases the efficiency of membrane disruption and is further evidenced in the time course of incubation at 37 °C, in which the MPA of N-EsxA dropped quickly over time, while the MPA of EsxAB remained stable (Figure 4 and Figure 5).

In summary, this new experimental model enabled us to characterize the membrane permeabilization of EsxAB at physiologically-relevant conditions. We conclude that anionic charged lipids are critical for EsxAB membrane binding followed by MPA and EsxB functions as a chaperone to stabilize N-EsxA at 37 °C prior to the acidification-driven heterodimer dissociation.
